# Gene expression, regulation of DEN and HBx induced HCC mice models and comparisons of tumor, para-tumor and normal tissues

**DOI:** 10.1186/s12885-017-3860-x

**Published:** 2017-12-18

**Authors:** Qin Tang, Qi Wang, Qiong Zhang, Sheng-Yan Lin, Yanhong Zhu, Xiangliang Yang, An-Yuan Guo

**Affiliations:** 1 Hubei Bioinformatics & Molecular Imaging Key Laboratory, Department of Bioinformatics and Systems Biology, Key Laboratory of Molecular Biophysics of the Ministry of Education, College of Life Science and Technology, Huazhong University of Science and Technology, Luoyu Road 1037, Wuhan, 430074 China; 2 National Engineering Research Center for Nanomedicine, College of Life Science and Technology, Huazhong University of Science and Technology, Wuhan, 430074 China

**Keywords:** Hepatocellular carcinoma - DEN - Hepatitis B - Mouse model - Gene expression - Regulatory network

## Abstract

**Background:**

Hepatocellular carcinoma (HCC) is the leading cause of cancer mortality. Chemical and virus induction are two major risk factors, however, the potential molecular mechanisms of their differences remain elusive. In this study, to identify the similarities and differences between chemical and virus induced HCC models, we compared the gene expression profiles between DEN and HBx mice models, as well as the differences among tumor, para-tumor and normal tissues.

**Methods:**

We sequenced both gene and microRNA (miRNA) expression for HCC tumor tissues, para-tumor and normal liver tissues from DEN model mice (30-week-old) and downloaded the corresponding microarray expression data of HBx model from GEO database. Then differentially expressed genes (DEGs), miRNAs and transcription factors (TFs) were detected by R packages and performed functional enrichment analysis. To explore the gene regulatory network in HCC models, miRNA and TF regulatory networks were constructed by target prediction.

**Results:**

For model comparison, although DEGs between tumor and normal tissues in DEN and HBx models only had a small part of overlapping, they shared common pathways including lipid metabolism, oxidation-reduction process and immune process. For tissue comparisons in each model, genes in oxidation-reduction process were down-regulated in tumor tissues and genes in inflammatory response showed the highest expression level in para-tumor tissues. Genes highly expressed in both tumor and para-tumor tissues in two models mainly participated in immune and inflammatory response. Genes expressed in HBx model were also involved in cell proliferation and cell migration etc. Network analysis revealed that several miRNAs such as miR-381-3p, miR-142a-3p, miR-214-3p and TFs such as Egr1, Atf3 and Klf4 were the core regulators in HCC.

**Conclusions:**

Through the comparative analyses, we found that para-tumor tissue is a highly inflammatory tissue while the tumor tissue is specific with both inflammatory and cancer signaling pathways. The DEN and HBx mice models have different gene expression pattern but shared pathways. This work will help to elucidate the molecular mechanisms underlying different HCC models.

**Electronic supplementary material:**

The online version of this article (doi: 10.1186/s12885-017-3860-x) contains supplementary material, which is available to authorized users.

## Background

Hepatocellular carcinoma (HCC) accounts for 85 - 90% of liver malignancies and is the second cause of cancer death in the world [1]. Although many progresses have been achieved on HCC research, it still needs a better understanding of the molecular and regulatory mechanisms involved in HCC [2]. HCC is often arisen by several risk factors including hepatitis virus B or C (HBV or HCV) infection, chemical damage and chronic excessive alcohol intake and so on [3]. The occurrence of HBV induced HCC attributes to HBV proliferation and DNA integration into host genome to initiate the malignant proliferation and transformation [4]. The HBx (HBV regulatory x protein) of hepatitis B virus plays a crucial role in hepatocarcinogenesis by transcriptional activation, driving deregulated cell cycle progression, modulation of apoptosis and inhibition of nucleotide excision repair of damaged cellular DNA [5]. While chemical induced HCC leads to high levels of DNA damage and fails to block the cell cycle before the damaged DNA repaired [6]. In recent years, chemical-induced and virus-induced HCC mouse models are widely used for the studies of pathogenesis and drug treatment of HCC [6, 7]. However, the differences of gene expression and regulation in these two HCC models have not been compared, which is an important issue for model selection in HCC studies.

Diethylnitrosamine (DEN), a DNA alkylating agent, is widely used to induce liver cancer in rodent model with high success rate and similarity to human HCC [8]. Recently, DEN-induced rodent HCC models have been used to investigate the pathogenesis, prevention and treatment of liver cancer, which include evaluating miRNA functions, exploring the antitumor effects of drugs and identifying biomarkers and therapeutic targets etc. [9, 10]. HBx is a 154-amino acid hepatitis B viral protein which controls the level of HBV replication [11]. As early as 1991, Kim, et al. constructed transgenic mouse model harboring HBx gene and firstly found this gene can specifically induce liver cancer [12]. Lu, et al. used HBx to induce HCC in transgenic male C57 mice and identified some common regulators in HCC [13]. Ye, et al. studied the synergistic function of Kras mutation and HBx in mice HCC [14].

In recent years, microRNAs (miRNAs) and transcription factors (TFs) have drawn extensive attention in cancer research. They play pivotal roles in proliferation, differentiation, invasion and metastasis of tumors. Many miRNAs were reported as important regulators in HCC. For example, miR-221 promotes human HCC development and its silencing contributes to suppressing tumor properties [15]. MiR-214 and miR-375 suppress the proliferation of HCC cells by directly targeting E2F3 and AEG-1 respectively [16, 17]. TFs are paramount regulators in controlling gene expression in living organisms [18]. For example, PPARs contribute to the pathogenesis in cell cycling, anti-inflammatory responses and apoptosis [19]. Aberrant high expression of STAT3 may promote HCC migration and invasion [20]. To further explore the molecular mechanisms in complex diseases, regulatory networks are widely studied. Our previous studies have revealed the miRNA and TF co-regulatory motifs are pervasive regulatory models in biological processes and diseases [18, 21–23]. Through network analysis, the complex regulatory relationships in diseases will be illuminated on systematic level and the key regulators may be identified.

In this study, we applied bioinformatic approaches to analyze the high-throughput and microarray data of DEN and HBx HCC mice models. We aim to analyze the gene expression features and core regulatory factors in HCC from these two models, and reveal the similarities and differences between them as well as compare gene expression profiles in tumor, para-tumor and normal tissues in each model.

## Methods

### Sequencing of DEN model and data collection of HBx model

All of the experimental mice were male and purchased from Hubei Research Center of Laboratory Animals (Wuhan, China) and in the C57 genetic background. To induce HCC in mice, we used DEN as the inductive agent. In this study, all mice were divided into two groups: DEN group and control group. In DEN group, mice were fed with basal diet every day and given DEN treatment at a dose of 165 mg per kg body weight in sesame oil through oral once a week for 10 weeks, then drug treatment were stopped and the mice were fed with diet only until 30 weeks. In control group, mice were fed with basal diet only until 30 weeks. In order to determine whether the liver tissues have suffered cancerous changes, histopathologic examinations were conducted via microscope (Additional file 1: Figure S1). All of the animal procedures were performed in accordance with the Ethics Committee on Animal Experimentation of the Huazhong University of Science and Technology (Wuhan, China) and the NIH Guide for the Care and Use of Laboratory Animals (8th edition, 2011).

To detect gene and miRNA expression, high-throughput sequencing technologies were used: RNA-seq for detection of expressed transcripts and small RNA-seq for detection of miRNAs respectively. Samples of tumor and para-tumor liver tissues were excised from the same lobe of the liver. For control group, RNA was isolated from liver samples obtained from age-matched healthy mice. Three biological replicates were sequenced for each group. For RNA-seq, ribosome RNA was removed first and pair-end 150 bp sequencing were carried out through Illumina Hiseq 3000, while for small RNA-seq, single-end 50 bp sequencing were performed with Illumina Hiseq 2500. All of the sequencing and data filtering works were done by Ribobio company (Guangzhou, China).

The microarray expression data of HBx model were downloaded from Gene Expression Omnibus (GEO) (http://www.ncbi.nlm.nih.gov/geo/, GEO accession: GSE15251). This model used Hepatitis B virus X antigen (HBx) to induce HCC in transgenic male C57 mice. The microarray samples of tumor, para-tumor and normal tissues were from the 16-month-old mice and the experimental conditions were similar with our DEN model.

### Gene expression and differential expression analysis among tumor, para-tumor, and normal tissues

In DEN model, RNA-seq reads were firstly quality-checked by fastqc software, then HISAT2 (version: 2.0.5) was used for mapping sequencing reads to mouse genome GRCm38, and StringTie (version: 1.2.2) was used to assemble the RNA-seq alignments into potential transcripts based on the reference sequences and calculate the abundance of transcripts [24]. The expressed levels were estimated as FPKM (the number of Fragments (reads) per kilobase of transcript per million mapped reads). The differentially expressed genes (DEGs) were identified using NOISeq [25] with thresholds FDR < 0.01 and |fold-change| ≥ 1.5. For small RNA-seq, firstly reads were aligned to the mouse genome, mouse miRNA and miRNA precursor data using Bowtie2 [26], then the expression of miRNAs were estimated as RPM (reads per million mapped reads). The differential expression of miRNAs were calculated by DEGseq [27] and edgeR [28], the threshold was also set as |fold change| ≥ 1.5 and required their RPM ≥ 20 in at least one tissue. Results of two methods were pooled together subsequently. In HBx model, DEGs were identified by NOIseq with FDR < 0.01 and |fold-change| ≥ 4.0.

The expression characteristics of two models were displayed by cumulative distribution function plot. The definition of cumulative distribution function is: F*x*(x) = P(X ≤ x), where the right-hand side represents the probability that the random variable X takes on a value less than or equal to x [29]. And the top 5% expressed genes were extracted for function enrichment and comparison. To survey the functions of genes, we used DAVID (https://david.ncifcrf.gov/tools.jsp) for GO (gene ontology) and KEGG (Kyoto Encyclopedia of Genes and Genomes) enrichment analysis. The top ranked enrichment results with high significant levels (*p*-value <0.01) were selected for discussion and presented by bubble plots using R package ‘ggplot2’. In the bubble plot, rich factor is calculated as the ratio of gene counts that mapped to a certain pathway and the total gene number of that pathway.

To reveal the gene expression difference between tumor and non-tumor tissues in two models, the pairwise comparisons of DEGs were carried out among tumor, para-tumor and normal tissue. Venn diagrams of DEGs were drawn and pathway enrichment in each model were implemented as described above.

### Gene function comparison between DEN model and HBx model

To further compare similarities and differences between two models, we firstly analyzed the similarities and differences between tumor tissue in DEN model and HBx model, and then focused on the para-tumor tissues of two models. The common pathways in two models were shown by bar charts, and the different pathways were exhibit by bubble plots. What’s more, heatmaps were used to recognize the gene expression patterns among three tissues.

### Identification of miRNA and TF targets and construction of regulatory networks

Networks were constructed using the differentially expressed data by reference to the collected validated data from several databases. For miRNA, we collected experimentally verified targets as described in our previous review paper [22]. The data mainly include the overlapped results from miRWalk2, miRecords4, miRTarbase6 and Tarbase7. TF targets were extracted from TRANSFAC database. The regulatory interactions between miRNA/TF and genes were obtained via Python script. Finally Cytoscape (Version 3.2.1) were used to visualize the networks.

## Results

### The cumulative distribution of genes in DEN and HBx models and functions of highly expressed genes in tumor

**Fig. 1:**
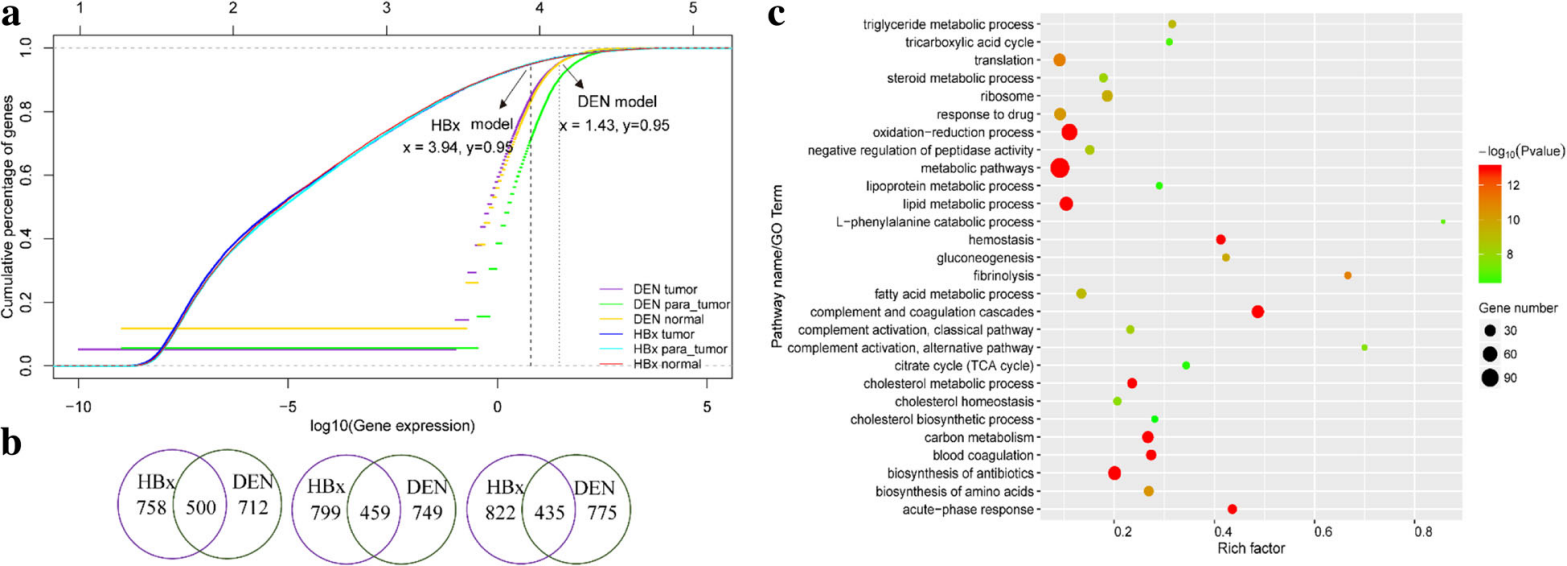
Gene expression summary and the highly expressed genes in both models. **a** The cumulative distribution of genes in DEN and HBx models. In this figure, the expression level of genes in DEN model is indicated by the bottom x-axis, while the expression level of gene in HBx model is indicated by the top x-axis, and y-axis indicate the cumulative percentage of genes. From left to right, gene expression level increases gradually, while from bottom to up the cumulative percentage of genes increases gradually. **b** Venn diagram of the top 5% highly expressed genes in DEN and HBx models. The left, middle, right diagram shows the overlap of high expressed genes in normal tissues, para-tumor tissues and tumor tissues respectively. **c** The function enrichment of highly expressed genes in HCC models (from tumor tissues)

In this study, a total of 20,901 genes were detected by high-throughput sequencing in DEN model, and 23,855 genes were identified in HBx model microarray data. To investigate the relationship between the increasing ratio of gene numbers and the gene expression level, the cumulative distributions of genes in two models are shown in Fig. 1a. In each model, three distribution curves represent different distributions of tumor, para-tumor and normal tissues respectively. The left-side of the dotted vertical line represents the cumulative distribution of 95% genes in that model, while the right-side represents the cumulative distribution of the top 5% high expressed genes. In DEN model, the log10 (FPKM) of 95% genes are less than 1.43 while in HBx model the log10 (fluorescence value) of 95% genes are less than 3.94. On the whole, these two models exhibit different characteristics of gene distribution: the distribution range of gene expression in DEN model is relatively narrow and sharp increasing, while the distribution range of gene expression in HBx model is relatively wide and slow increasing. This may due to the difference of gene expression platform (RNA-seq and microarray).

To investigate the functions of the key genes in two models, the comparison of top 5% highly expressed genes of the corresponding tissues in two models were carried out (Fig. 1b). The two models have an overlap of 500, 459 and 435 highly expressed genes in normal, para-tumor and tumor tissues respectively. Genes in tumor tissues may be directly related to the pathogenesis of HCC, so more attention was paid to the comparison of these genes in two models. Through biology process and KEGG pathway enrichment analysis, the common and different functions were identified. The top five shared processes and pathways were complement and coagulation cascades, acute-phase response, hemostasis, oxidation-reduction process and metabolism especially lipid metabolism (Fig. 1c). The top ranked specific pathways in tumor tissues of DEN model are oxidative phosphorylation, non-alcoholic fatty liver disease (NAFLD), antigen processing and presentation of exogenous peptide antigen. While in HBx model, sterol biosynthetic process, cell-cell adhesion and triglyceride homeostasis were obtained (Additional file 2).

### Gene expression difference among tumor, para-tumor, and normal tissues in both models

**Fig. 2:**
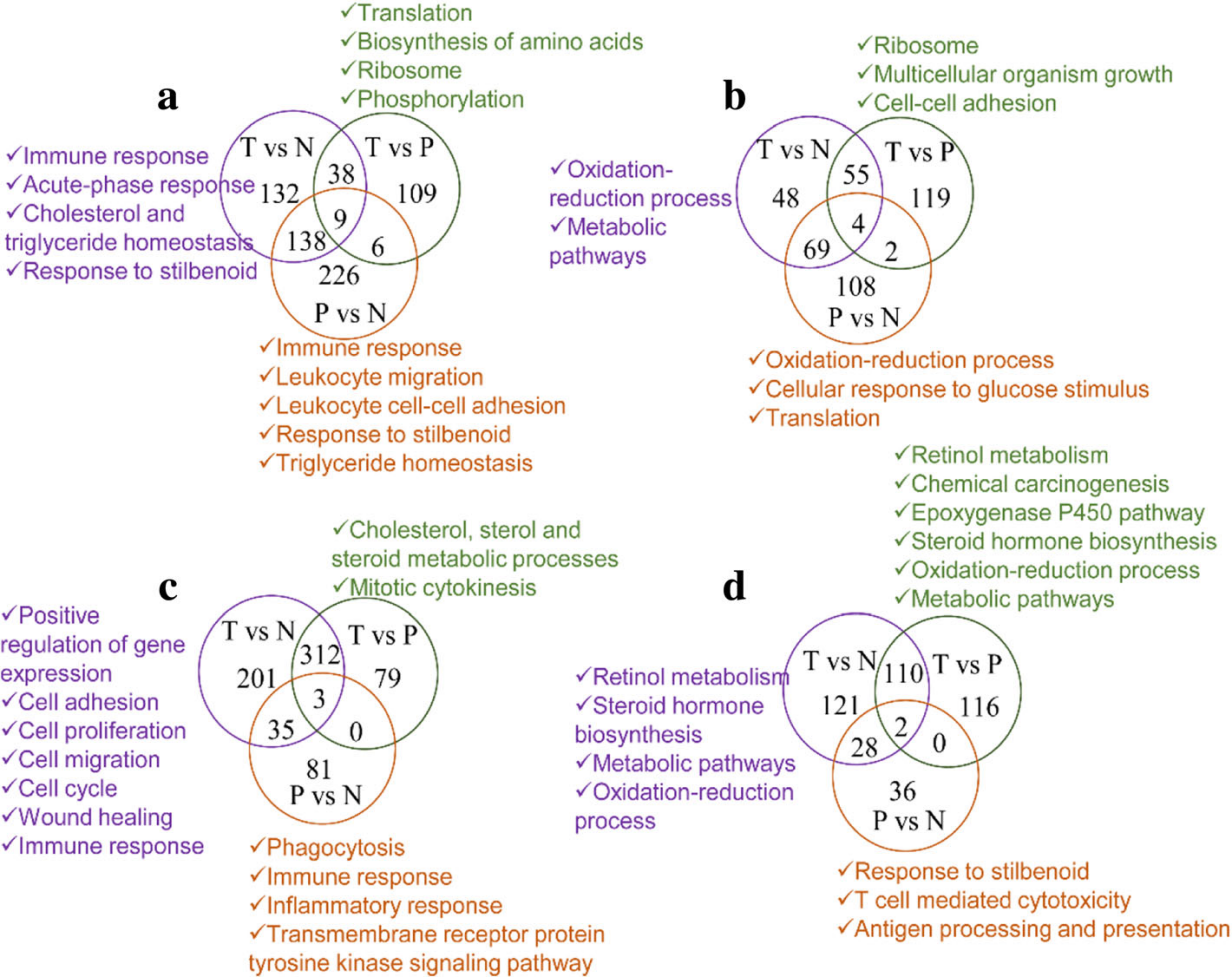
Venn graphs of DEGs in DEN model and HBx model. The alphabets T, P and N represent tumor, para-tumor and normal tissue respectively. **a** Venn graph of up-regulated genes in DEN model. **b** Venn graph of down-regulated transcripts in DEN model. **c** Venn graph of up-regulated genes in HBx model. **d** Venn graph of down regulated genes in HBx model. The main functions of up- or down-regulated genes are labeled beside and marked by the same color with the loop

To compare the gene expression between tumor and non-tumor tissues in two models, the pairwise comparisons of DEGs were performed among tumor, para-tumor and normal tissues. The statistical results of up- and down-regulated genes in each model were shown in Fig. 2. Firstly, we focused on the functions of up- and down-regulated DEGs in tumor vs normal tissues in two models. The comparison results revealed that the up-regulated genes of tumor vs normal tissue in DEN model were mainly involved in immune response, acute-phase response, cholesterol and triglyceride homeostasis (Fig. 2a). While genes in down-regulated group of DEN model were involved in oxidation-reduction process, metabolic pathways such as cholesterol and steroid metabolic process (Fig. 2b). In HBx model, the up-regulated genes of tumor vs normal tissue were (Fig. 2c) mainly involved in positive regulation of gene expression, cell adhesion, cell proliferation, cell migration, cell cycle, wound healing, immune response. But in down-regulated group of HBx model (Fig. 2d), genes were also involved in metabolic pathways and oxidation-reduction process, which are similar to DEN model. For the comparison between para-tumor and normal tissue, up-regulated genes in para-tumor in both models were enriched in immune and inflammatory response which suggested the para-tumor tissues were in high inflammatory status. The functional enrichment results of other comparisons were also shown in Fig. 2.

**Fig. 3:**
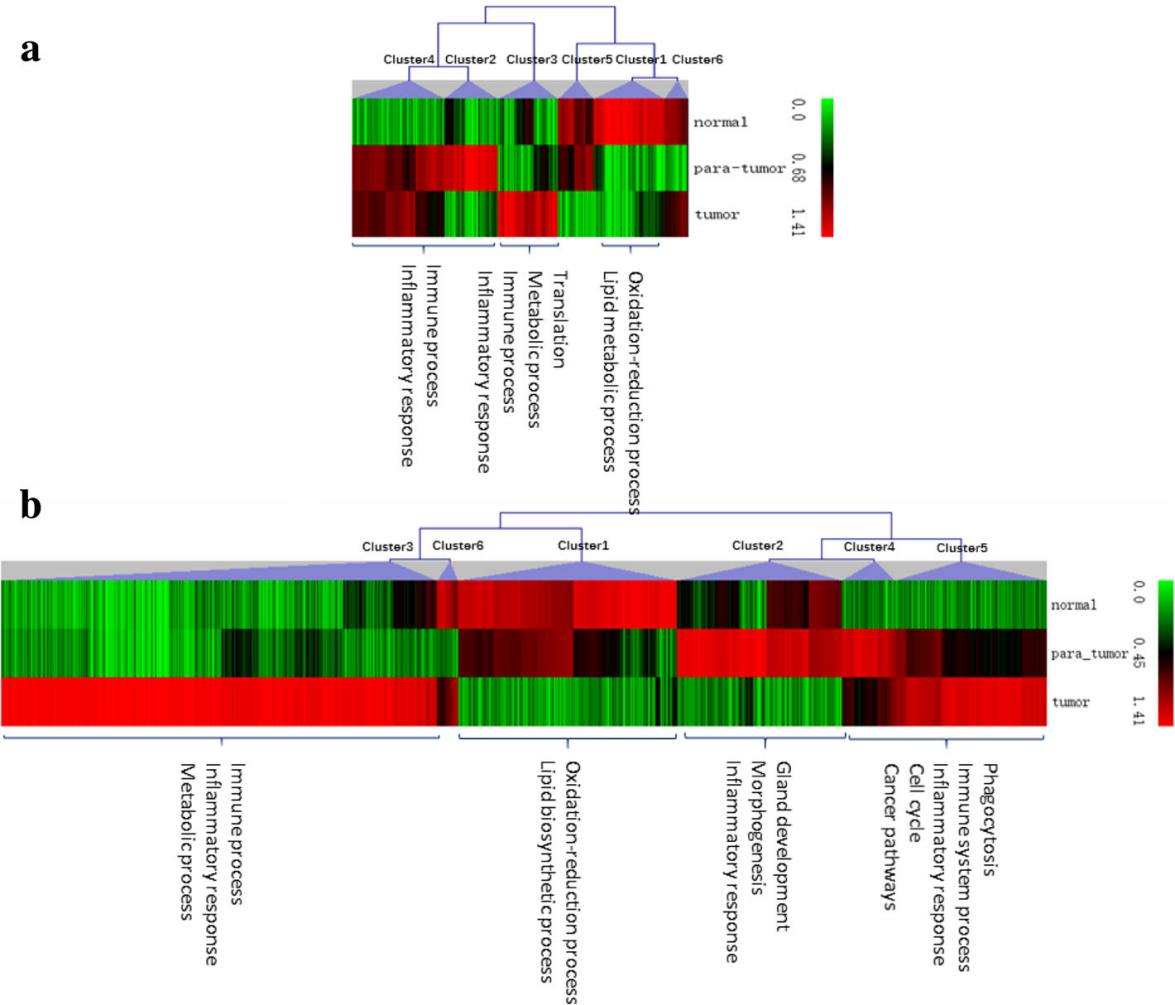
Clusters and main functions of DEGs in DEN model (**a**) and HBx model (**b**)

To further investigate gene expression differences among three tissues in two models, we recognized the gene expression patterns and found 6 clusters in each model (Fig. 3). Genes with highest expression in normal tissue (Cluster1 in Fig. 3a and b) mainly involved in oxidation-reduction process and lipid metabolic process. Genes in Cluster2 of Fig. 3a and b were in the highest expression in para-tumor tissue. The common function of these genes in both models is inflammatory response, while the specific function in DEN model includes immune process and in HBx model concludes gland development and morphogenesis. The pattern of the highest expression in tumor tissue were recognized in Cluster3 of Fig. 3a and b. The same functions of this pattern in two models include immune process, inflammatory response and metabolic process especially sterol, cholesterol and steroid metabolism. What’s more, we also focused on the genes highly expressed in both tumor and para-tumor tissues (Cluster4 in Fig. 3a, Cluster4 and Cluster5 in Fig. 3b), these genes mainly participated in immune system process, inflammatory response, phagocytosis, cell cycle and several cancer pathways.

From the above results, we could summarize that the healthy liver tissue is mainly characterized with lipid biosynthetic and metabolic process and oxidation-reduction on account of maintenance the normal life activities, while the tumor tissue is characterized with immune, inflammatory response and cancer pathways. The immune effect and inflammatory response in para-tumor tissue are higher than normal tissue. What’s more, these responses also show a slight declined trend in tumor tissue when compared with para-tumor tissues. Therefore, we suggest that the para-tumor tissue should be regarded as inflammatory tissue rather than normal tissue.

### Functional comparison of DEGs between DEN and HBx models

**Fig. 4:**
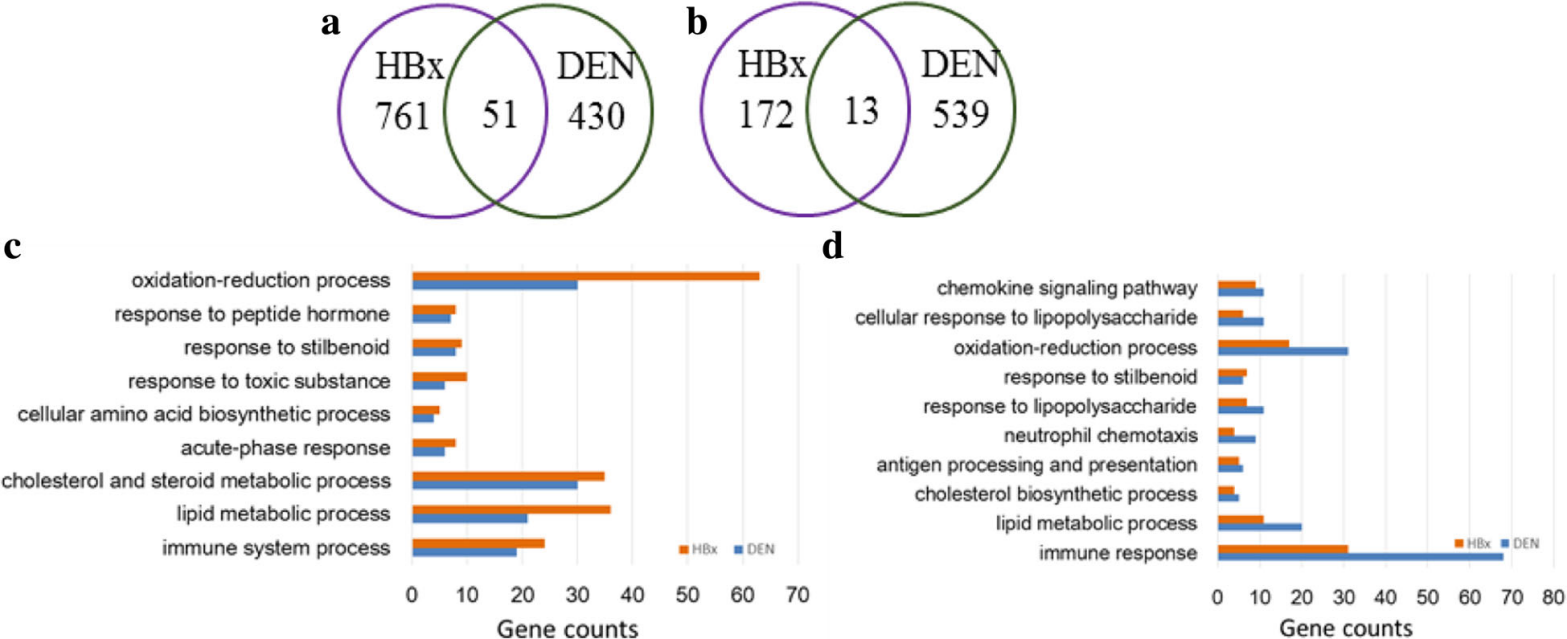
The comparisons and functions of DEGs in two models. **a** The comparison of DEGs between tumor tissues (use tumor-vs-normal as tumor specifically expressed genes). **b** The comparison of DEGs between para-tumor tissues (use para-tumor-vs-normal as para-tumor genes). **c** Common pathways of tumor tissue in two models. **d** Common pathways of para-tumor tissue in two models

To disclose the similarities and differences of gene functions between two models, we further focused on tumor tissues and para-tumor tissues in two models. The DEGs of tumor vs normal tissue and para-tumor vs normal tissue were regarded as the specific genes of tumor tissue and para-tumor tissue, respectively. As a result, we found the DEN and HBx models only shared a small part of tumor specific genes (Fig. 4a). Further, we compared their gene functions and found 9 common functional terms (Fig. 4c). The top ranked common pathways of tumor tissues were oxidation-reduction process, metabolic processes especially lipid, cholesterol and steroid metabolic processes. There are also dozens of different enriched pathways in DEN and HBx models (Additional file 1: Figure S2 a). In tumor tissue of HBx model, the most significant different pathways are retinol metabolism, wound healing, positive regulation of gene expression and positive regulation of protein kinase B signaling, while in DEN model, the most significant different pathway is chemical carcinogenesis. Although only a few para-tumor genes shared by two models (Fig. 4b), functional enrichment analysis indicated 10 shared pathways (Fig. 4d), top of which is immune response. There are also dozens of different enriched pathways (Additional file 1: Figure S2 b). In para-tumor tissue of HBx model, the most significant different pathway is phagocytosis, while in DEN model the most significant different pathways are response to interferon-gamma and the negative regulation of neuron death.

### Differentially expressed miRNAs and its regulatory network in DEN model

**Fig. 5:**
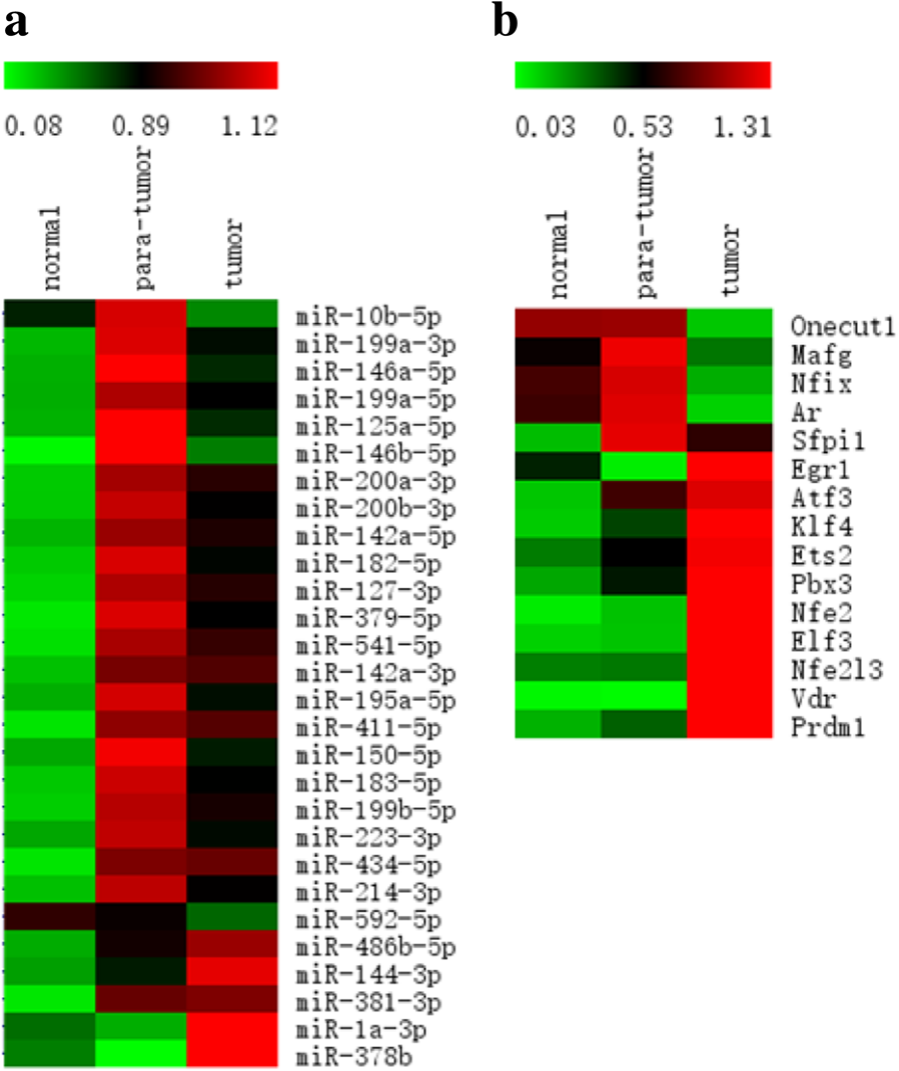
The expression of differentially and highly expressed miRNAs and TFs. **a** Differentially and highly expressed miRNAs in three tissues of DEN model. **b** Differentially and highly expressed TFs in three tissues of HBx model

To explore the miRNA expression and their regulation to genes in HCC, we sequenced the miRNA in the DEN model. We totally obtained 28 differentially and highly expressed miRNAs (Fig. 5a) from the pairwise comparisons of three tissues. Most of the miRNAs showed the highest expression level in para-tumor tissue and the lowest level in normal tissue, but their differences were not significant by comparing tumor and para-tumor tissues. To explain this phenomenon we conducted immunohistochemistry experiments to quantify the proteins of two miRNA produced genes, DROSHA and ADAR. The results showed that the highest expression of these two proteins were shown in para-tumor tissue, while the lowest expression level indicated in normal tissue (Additional file 1: Figure S3 a), and their variation trends were consistent with the RNA-seq data (Additional file 1: Figure S3 b). The top ten highly expressed miRNAs (ranked by the mean expression value of three tissues) are miR-199a-3p, miR-199a-5p, miR-146a-5p, miR-146b-5p, miR-125a-5p, miR-200a-3p, miR-200b-3p, miR-142a-5p, miR-486b-5p and miR-182-5p.

**Fig. 6:**
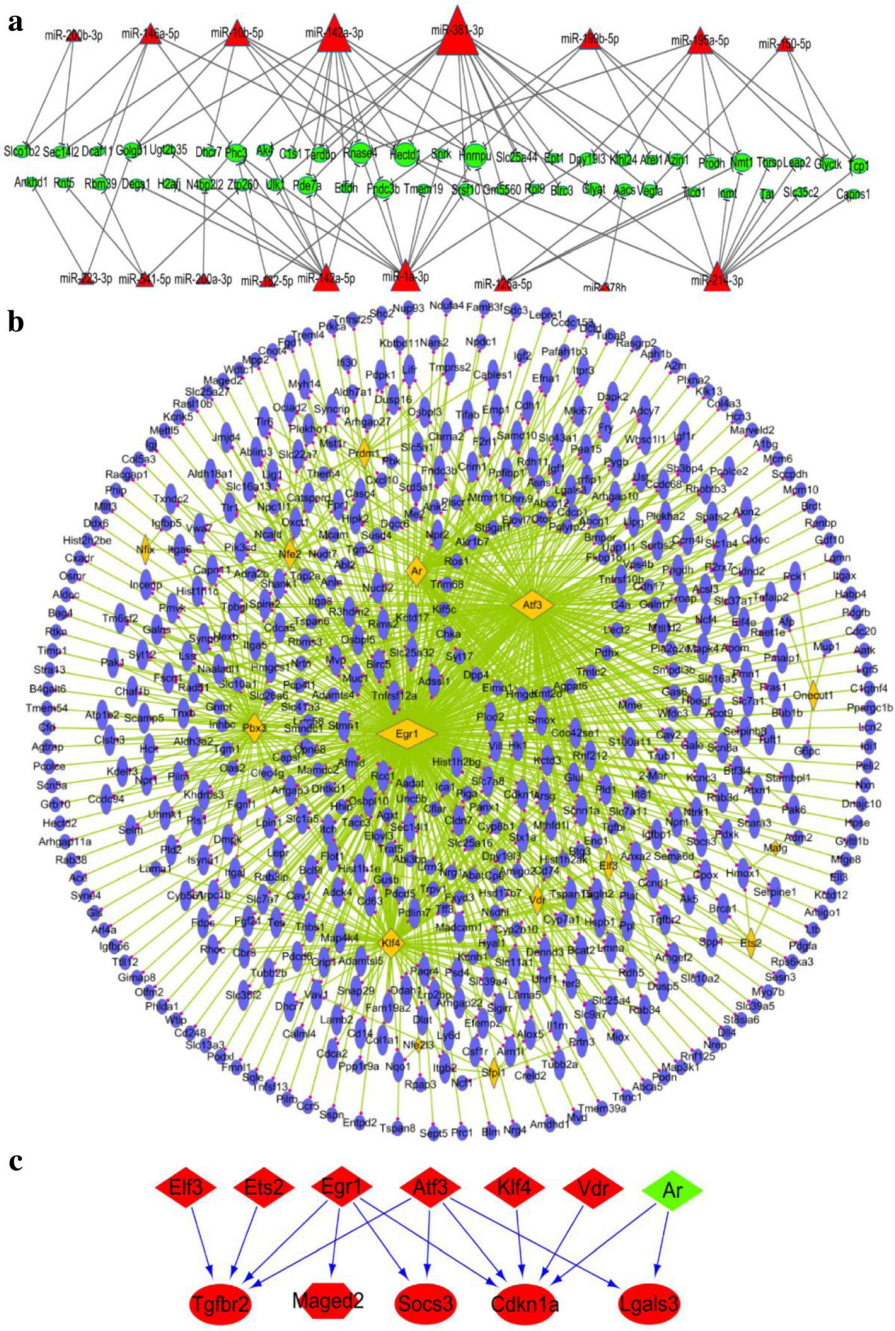
The miRNA and TF regulatory networks in DEN and HBx model, respectively. **a** The regulation of differentially expressed miRNAs to target genes in DEN model. Triangle: miRNAs. Oval: genes. **b** The regulation of differentially expressed TFs to target genes in HBx model. Gold diamond: TFs. Purple oval: genes. The node size is positively related to the node degree. **c** The regulation of differentially expressed TFs to significant oncogenes and tumor supressor genes. Colors: red represents up-regulated in tumor tissue than in normal tissue; green represents down-regulated in tumor tissue. Triangle: TFs. Oval: tumor supressor genes. Hexagon: Oncogene

To recognize the molecular regulation mechanism in HCC, a miRNA-gene regulation network was constructed in DEN model. Firstly, we found out a total of 186 genes that show opposite expression tendency with miRNAs. Then regulatory relationships between the miRNAs and genes were screened out according to verified miRNA targets which collected from several databases. Finally 98 regulatory pairs were obtained and intergrated into a network containing 17 miRNAs and 50 genes with 98 edges (Fig. 6a). In this network, the top three significant miRNAs with most targets were miR-381-3p, miR-142a-3p, miR-214-3p, which may be the important regulatory factors in DEN HCC. It was reported that miR-381 was up-regulated in mouse liver and acted as a hub regulators [30], miR-142-3p can suppresses the migration and invasion of HCC cells by regulating RAC1 [31], and miR-214 can suppress invasion, stem-like traits and recurrence of HCC through targeting beta-catenin pathway [32]. Our results were not only in accordance with these reports, but also revealed some synergistic regulations.

### Differentially expressed TFs and its regulatory network in HBx model

For the HBx model, although we have no corresponding miRNA data, we can identify the differnetially expressed TFs and explore the TF regulatory network. From the DEGs we obtained 15 highly expressed TFs (Fig. 5b). The regulatory relationships between 15 TFs and 524 genes were screened out and presented in a network (Fig. 6b). This network included a total of 539 nodes and 938 edges. Obviously, it is creditable to recongize that the TFs Egr1, Atf3 and Klf4 are the main regulatory factors in this model. The importance of these TFs have been verified by previous studies. It was reported that EGR-1 can inhibit the growth of hepatocellular carcinoma cell lines and suppress the transformation activity in fibrosarcoma and breast carcinoma [33], ATF3 and KLF4 were reported as tumor suppressors in HCC [34, 35]. Subnetworks of these TFs are listed in Additional file 1: Figure S4. Through enrichment analysis, we found that the targets of these TFs mainly participated in cell adhesion, proliferation, migration, apoptotic and multiple cancer signaling pathways such as PI3K-Akt, MAPK, Ras and p53 signalling pathway, which are important pathways in HCC.

To investigate the synergistic regulations on oncogenes and tumor suppressor genes (TSGs) of TFs, we collected the oncogenes and TSGs in HCC from previous publications [36, 37]. We found that 5 of these genes were in the TF regulatory network (Fig. 6b) and a sub-network containing the 5 genes and 7 TFs was extracted to exhibit the synergistic regulations (Fig. 6c). From the network, we found both the TSG Tgfbr2 and Socs3 can be co-regulated by tumor suppressor Egr1 and Atf3, while the TSG Cdkn1a is the most significant gene which can be co-regulated by 5 TFs (Egr1, Atf3, Klf4, Vdr and Ar). TSG Lgals3 can be co-regulated by Atf3 and Ar.

## Discussion

In this study, we investigated the highly expressed genes in both DEN and HBx mice models, and identified the DEGs through comparison among tumor, para-tumor and normal tissues. The gene expression profiles and function enrichment of different tissues demonstrate different characteristics and functional specificities of three tissues. Moreover, through comparison of two models, we found both similarities and differences exited in gene expression. Finally, we constructed miRNA and TF regulatory networks and identified several key regulators from the networks, as well as revealed some synergistic regulations in HCC models.

Through comparison of tumor, para-tumor and normal tissues, we suggest that para-tumor liver tissues should be regarded as inflammatory tissue rather than normal tissue. But in the study of human solid carcinomas, due to the sampling limitations, researchers usually use comparison of tumor and adjacent non-tumor tissues of patients to illustrate the molecular mechanism of cancers. In fact, from our study, the maximal effects of immune responses and inflammatory responses were detected in para-tumor tissues rather than tumor tissues or normal tissues. While the normal tissues were characterized with lipid mechanism and oxidation-reduction, and the tumor tissues enriched in cancer signal transduction, immune and inflammatory responses (Figs. 2 and 3). Till to now, only a few articles have reported the comparisons among three tissues. Chandran et al. investigated the gene expression differences among prostate cancer tissue, adjacent prostate cancer tissue and normal prostate tissue from cancer free organ donors. They found that the tumor vs normal expression profile was more extensive than the tumor vs adjacent normal profile, when using normal as the baseline, similar genes were up-regulated in both tumor and adjacent tissue, and these genes can not be identified from tumor vs adjacent normal profile [38]. In other words, para-tumor tissue is somewhat similar to tumor tissue. Also in this study, the tumor and para-tumor tissues emerged with higher response of inflammatory and immune than normal tissues, which was in agreement with previous report that inflammation was closely related to cancer. Grivennikov and Karin reported that about 20% of all cancers generated in association with chronic inflammation, and most of the solid tumors contain proinflammatory cytokines which can mediate immune responses and effect tumor initiation, growth and progression [39].

In previous studies, both DEN- and HBx-induced mice liver cancer models have been used to investigate the pathogenesis of HCC, they have become the two major concerns in liver cancer research [9, 10, 13]. It has been verified that the cancers of DEN and HBx induced in mice are very similar to human HCC [8, 40]. Comparison of these two representative models can accurately reveal the mechanism of HCC carcinogenesis. But so far, no studies have investigated the difference of molecular mechanisms between drug-induced and virus-induced HCC. Through comparison of the same tissues between two models, we found that immune response, inflammatory responses, metabolic process, and oxidation-reduction process are the common activities in both two models. There are also several different enriched pathways in two models. The most significant different pathway in DEN model is chemical carcinogenesis while in HBx model they are retinol metabolism, wound healing, positive regulation of gene expression and positive regulation of protein kinase B signaling pathway. These differences may due to the different induction of two model (DEN and HBx). Another obviously difference is that the HBx model appears with more cancer related activities, for example, cell signal transduction and communication, cell cycle and programmed cell death. This may due to the long/short period of induction. DEN induction is more acute and can resulted in serious injury even death before all the cancer pathways significantly emerged, while HBx induction is chronic and mice can live longer until all the cancer pathways obviously emerged. It was reported that signaling molecules such as EGFR, Ras, PKC, AKT/PKB and mTOR, were found to play important roles in human cancer cells and were involved in cell proliferation, differentiation and survival [41]. PDCD4 (Programmed Cell Death Protein4) gene can be inhibited by miRNA-21 and results in the failure of programmed cell death, and finally enhances cell proliferation in HCC and breast cancer [42, 43]. These molecules or their subtypes were also found highly expressed in the models in this study.

At the same time, we also identified differentially expressed miRNAs and TFs from the data, and constructed two major regulatory networks. From network analysis, miR-381-3p, miR-142a-3p, miR-214-3p and Egr1, Atf3, Klf4 were identified to be the most important regulators in HCC. These are important tumor suppressors which can be considered as diagnosis biomarkers and treatment targets. What’s more, the synergistic regulation analysis indicated that Cdkn1a is the most import target gene of multiple TFs in HCC (Fig. 6c). It can encode a potent cyclin-dependent kinase inhibitor and inhibits the activities of several cyclin-cyclin-dependent kinases, not only functions as a regulator of cell cycle progression at the G1 pahse, but also involved in the regulation of transcription, apoptosis, DNA repair and cell motility [44]. Previous study has reported that the expression of this gene is tightly controlled by the tumor suppressor protein p53 [45]. In this study, we found it was also synchronously regulated by the TFs Egr1, Atf3, Klf4, Vdr and Ar, this result enriched the recognition of regulatory mechanisms of this gene. Besides, the TSG Tgfbr2 is also a top ranked gene that was synchronously regulated by multiple TFs. Tgfbr2 is a key molecule in TGFbeta signaling pathway and was found to prevent the formation of hepatocellular carcinomas and cholangiocarcinoma development [46]. Taken together, through network analysis we identified several pivotal regulators and cancer related genes, this work provided comprehensive information for gene regulation involved in HCC.

## Conclusions

Through gene expression and function analyses, we found that both DEN and HBx model shared common pathways including lipid metabolism, oxidation-reduction process, immune process and inflammatory responses. Through tissue comparison, we suggest that the para-tumor should be recognized as inflammatory tissue. Network analysis revealed that miRNAs such as miR-381-3p, miR-142a-3p, miR-214-3p and TFs such as Egr1, Atf3 and Klf4 are the hub regulators in HCC. These results will help to increase understanding of molecular mechanisms of HCC.

## Additional files

Additional file 1: Figure S1. Histopathologic examinations of liver tissues under microscope. All the pictures were captured at magnification of 100×. The first two lines are tissue slices of control and DEN treatments at different time point in DEN model. The third line indicates that the Kupffer cells increased over time. Kupffer cell is a kind of specialized macrophage which plays a major anti-inflamination role in liver, its increasing can reflect the injury level of liver. In this study, the injury significantly increased as time goes on. The pictures in the last line show histological changes from control to para-tumor and tumor tissues of liver at the 30th week. **Figure S2.** Different pathways in the same tissue in two models. **a** Tumor tissues. **b** Para-tumor tissues. The terms on vertical axis beginning with ‘DEN’ or ‘HBx’ represent the enrichment terms of genes in DEN model or HBx model. **Figure S3.** Quantification of the proteins of two genes, DROSHA and ADAR. (a) Pictures of immunohistochemistry results. (b) Gene expression levels (FPKM) and protein expression levels (Integrated optical density, calculated by Image pro plus 6.0) of these two genes. **Figure S4.** The subnetworks of the TFs Egr1, Atf3 and Klf4. Gold diamond: TFs. Purple oval: genes. (DOCX 7118 kb)

Additional file 2: Gene enrichment analysis of top 5% highly expressed genes of tumor tissue in DEN model and HBx model. (XLSX 57 kb)

## Abbreviations

DEGs: Differentially expressed genes
DEN: Diethylnitrosamine
HBx: Hepatitis B virus X antigen
HCC: Hepatocellular carcinoma
miRNA: MicroRNA
TF: Transcription factor
