# Supplementary material for: Gene expression, regulation of DEN and HBx induced HCC mice models and comparisons of tumor, para-tumor and normal tissues
Source: BMC Cancer. 2017 Dec 18;17:862. doi: 10.1186/s12885-017-3860-x (PMC5735680; doi:10.1186/s12885-017-3860-x)
Supplement: Supplementary file 1 — Histopathologic examinations of liver tissues under microscope. All the pictures were captured at magnification of 100×. The first two lines are tissue slices of control and DEN treatments at different time point in DEN model. The third line indicates that the Kupffer cells increased over time. Kupffer cell is a kind of specialized macrophage which plays a major anti-inflamination role in liver, its increasing can reflect the injury level of liver. In this study, the injury significantly increased as time goes on. The pictures in the last line show histological changes from control to para-tumor and tumor tissues of liver at the 30th week. Figure S2. Different pathways in the same tissue in two models. a Tumor tissues. b Para-tumor tissues. The terms on vertical axis beginning with ‘DEN’ or ‘HBx’ represent the enrichment terms of genes in DEN model or HBx model. Figure S3. Quantification of the proteins of two genes, DROSHA and ADAR. (a) Pictures of immunohistochemistry results. (b) Gene expression levels (FPKM) and protein expression levels (Integrated optical density, calculated by Image pro plus 6.0) of these two genes. Figure S4. The subnetworks of the TFs Egr1, Atf3 and Klf4. Gold diamond: TFs. Purple oval: genes. (DOCX 7118 kb) [file 12885_2017_3860_MOESM1_ESM.docx]

**
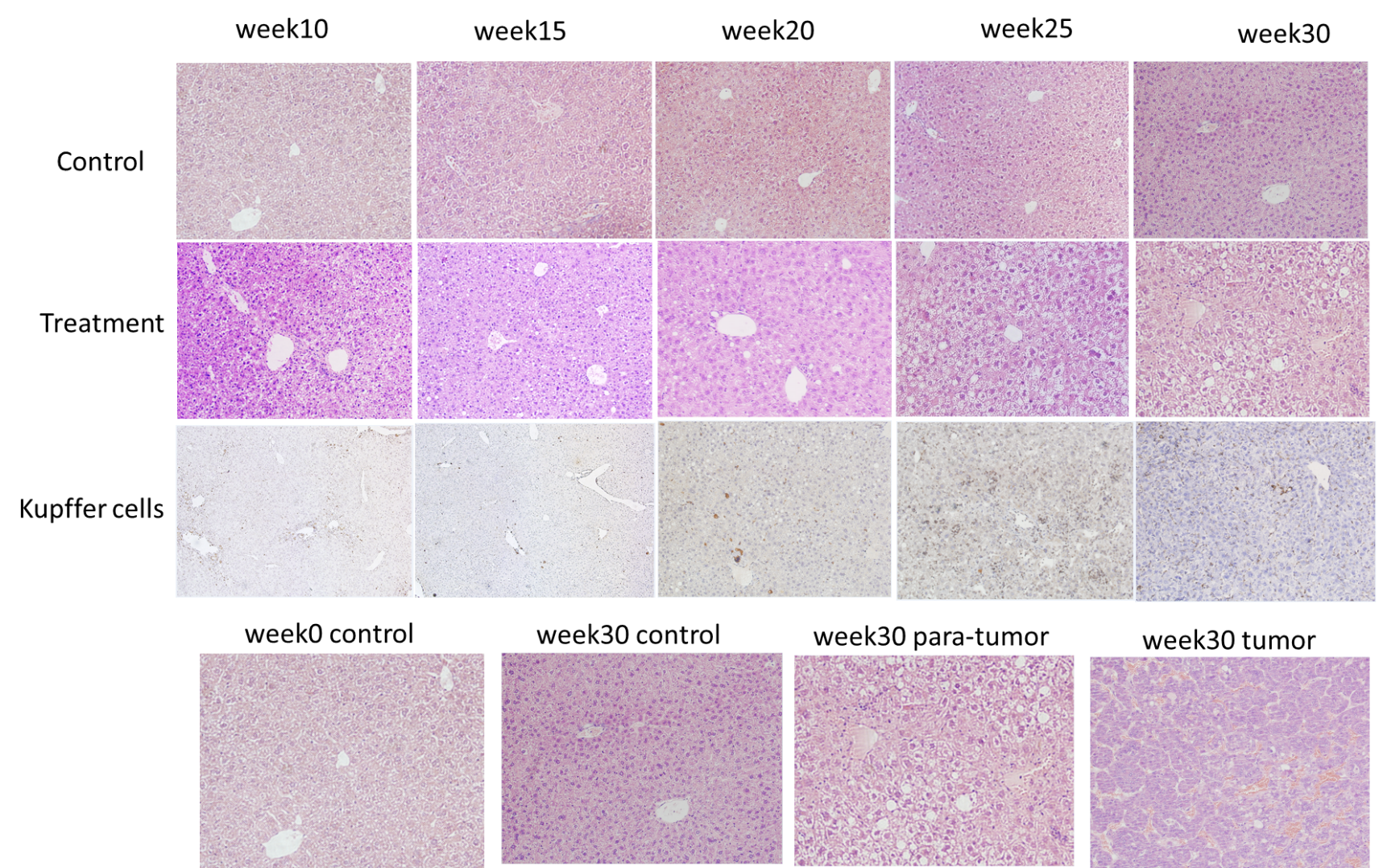
**

**Figure S1.** Histopathologic examinations of liver tissues under microscope.

All the pictures were captured at magnification of 100x. The first two lines are tissue slices of control and DEN treatments at different time point in DEN model. The third line indicates that the Kupffer cells increased over time. Kupffer cell is a kind of specialized macrophage which plays a major anti-inflamination role in liver, its increasing can reflect the injury level of liver. In this study, the injury significantly increased as time goes on. The pictures in the last line show histological changes from control to para-tumor and tumor tissues of liver at the 30th week.


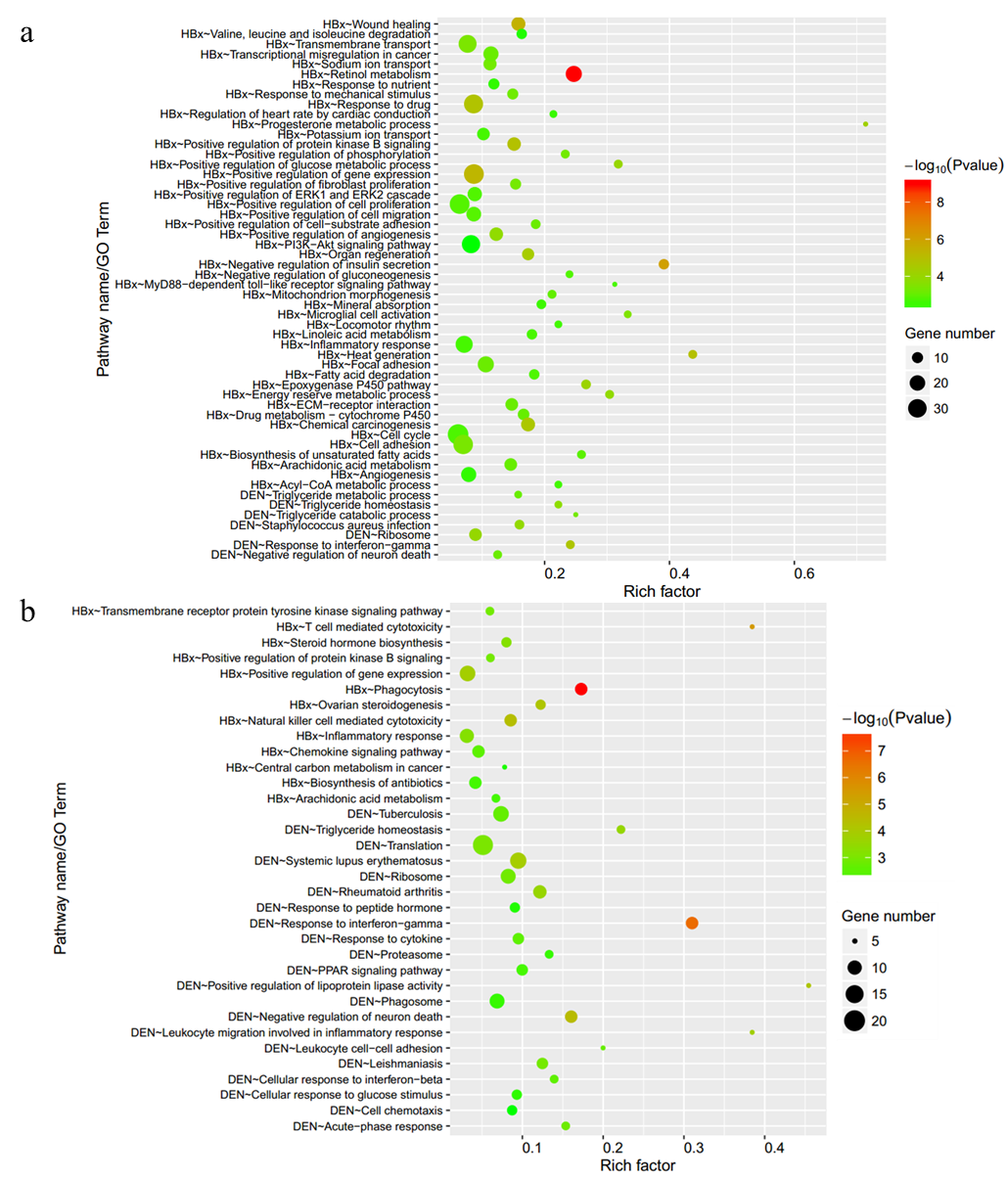


**Figure S2.** Different pathways in the same tissue in two models.

**(a)** tumor tissues **(b)** para-tumor tissues. The terms on vertical axis beginning with ‘DEN’ or ‘HBx’ represent the enrichment terms of genes in DEN model or HBx model.


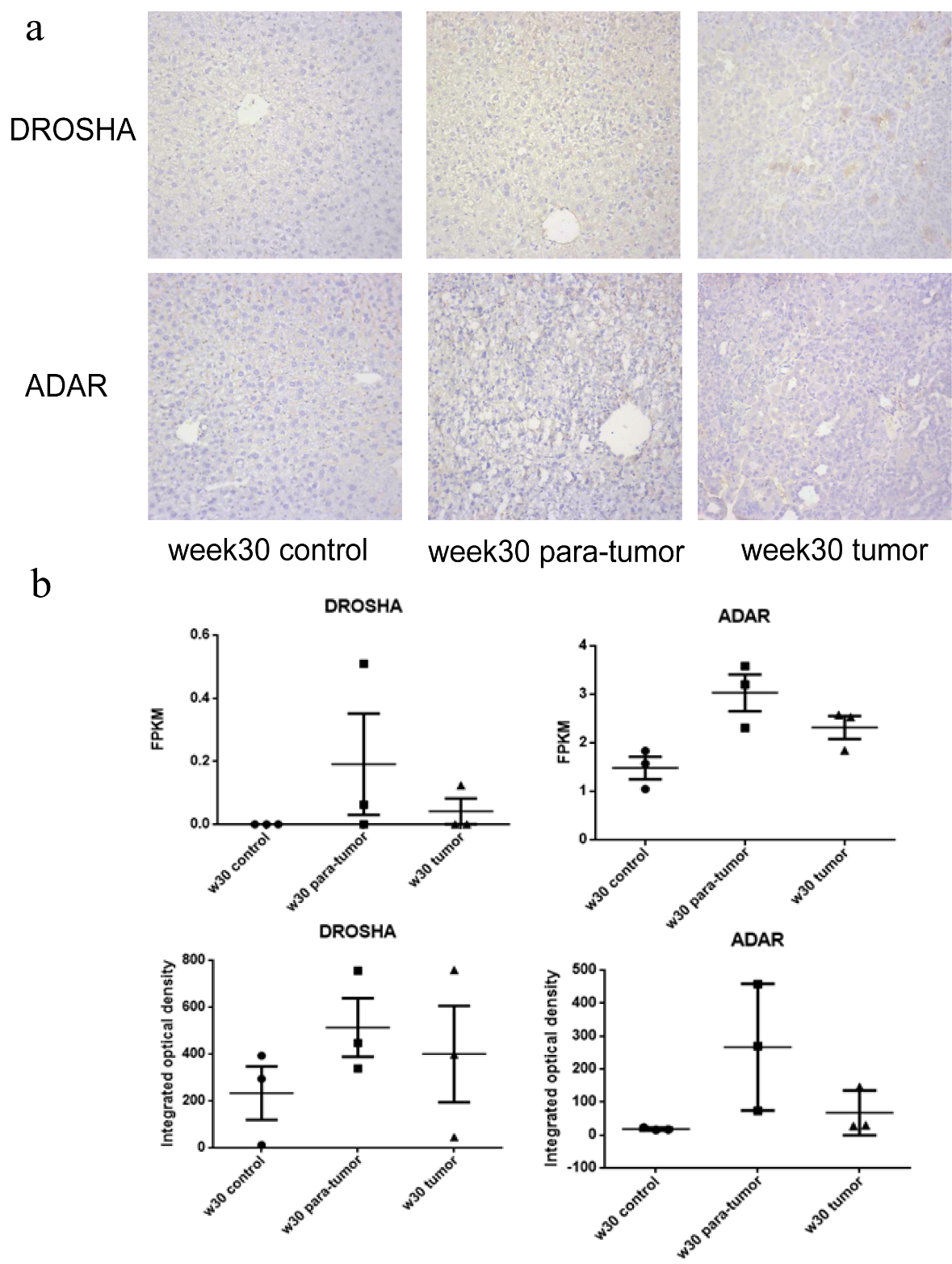


**Figure S3.** Quantification of the proteins of two genes, DROSHA and ADAR.

**(a)** Pictures of immunohistochemistry results. **(b)** Gene expression levels (FPKM) and protein expression levels (Integrated optical density, calculated by Image pro plus 6.0) of these two genes.

**
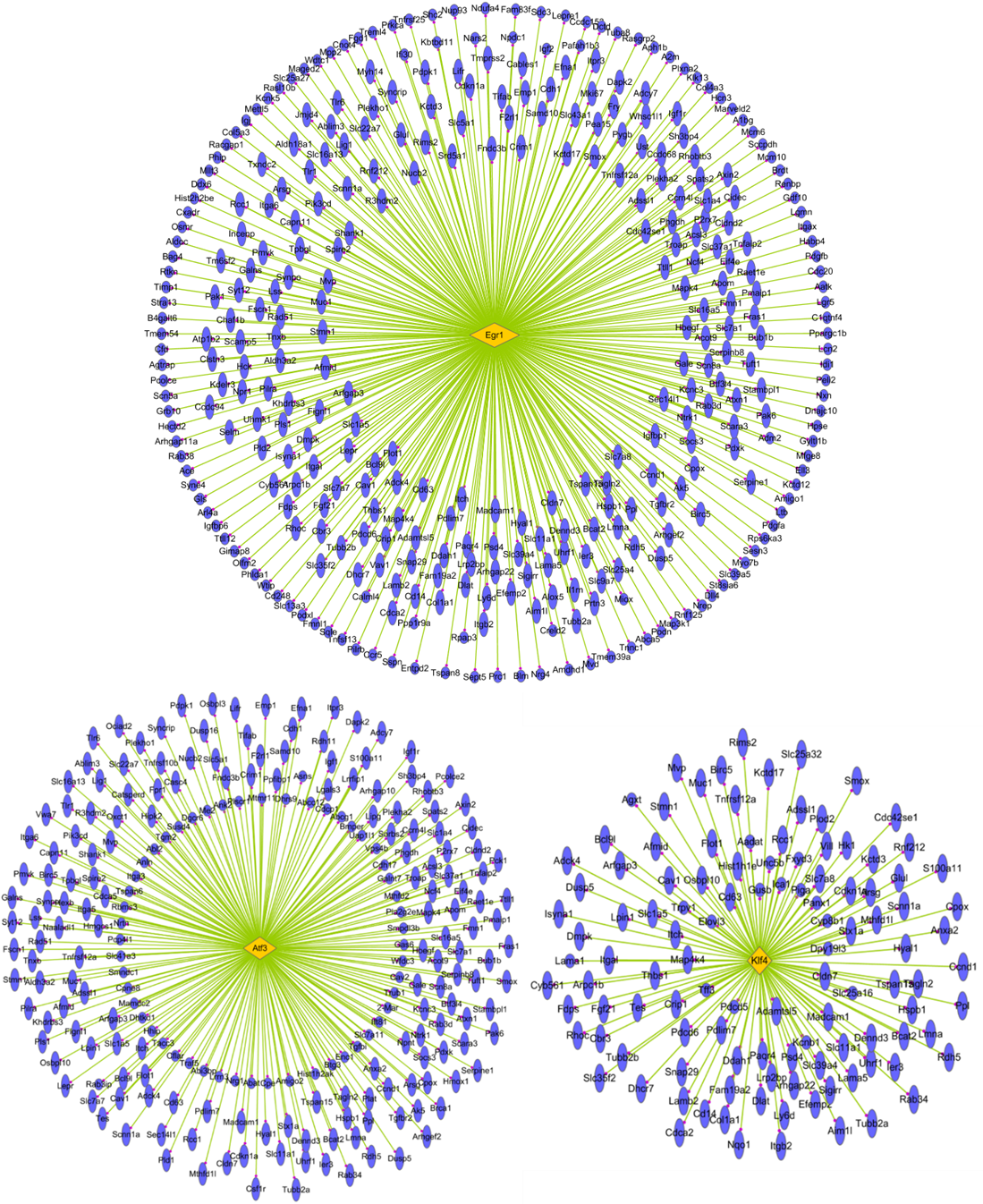
**

**Figure S4.** The subnetworks of the TFs Egr1, Atf3 and Klf4. Gold diamond: TFs. Purple oval: genes.
